# Creation of vortices by torque in multidimensional media with inhomogeneous defocusing nonlinearity

**DOI:** 10.1038/srep09420

**Published:** 2015-03-24

**Authors:** Rodislav Driben, Torsten Meier, Boris A. Malomed

**Affiliations:** 1 Department of Physics & CeOPP, University of Paderborn, Warburger Straβe 100, D-33098 Paderborn, Germany; 2 ITMO University, 49 Kronverskii Ave., St. Petersburg 197101, Russian Federation; 3 Department of Physical Electronics, School of Electrical Engineering, Faculty of Engineering, Tel Aviv University, Tel Aviv 69978, Israel

## Abstract

Recently, a new class of nonlinear systems was introduced, in which the self-trapping of fundamental and vortical *localized* modes in space of dimension *D* is supported by cubic *self-repulsion* with a strength growing as a function of the distance from the center, *r*, at any rate faster that *r^D^*. These systems support robust 2D and 3D modes which either do not exist or are unstable in other nonlinear systems. Here we demonstrate a possibility to create solitary vortices in this setting by applying a phase-imprinting torque to the ground state. Initially, a strong torque completely destroys the ground state. However, contrary to usual systems, where the destruction is irreversible, the present ones demonstrate a rapid restabilization and the creation of one or several shifted vortices orbiting the center. For the sake of comparison, we show analytically that, in the linear system with a 3D trapping potential, the action of a torque on the ground state is inefficient and creates only even-vorticity states with a small probability.

The theoretical prediction and experimental creation of multidimensional (two- and three-dimensional, 2D and 3D) solitons, i.e., self-trapped field patterns, is a challenging problem of nonlinear physics, which is especially relevant to Bose-Einstein condensates (BECs) and photonics, where such self-trapped states have the meaning of spatiotemporal solitons^1^,^2^,^3^,^4^. A serious problem is posed by the well-known fact that fundamental multidimensional localized states, supported by the generic cubic self-focusing nonlinearity, are subject to instability caused by the collapse of the self-attracting field^5^,^6^,^7^,^8^,^9^. Under the same conditions, more complex multidimensional localized states, in the form of *vortex tori*, alias vortical solitons, are vulnerable to a still stronger azimuthal instability, which splits them into fragments, that later suffer the intrinsic collapse^2^,^4^,^10^. Nevertheless, it was very recently found^11^ that *stable* complexes, composed of fundamental and vortex solitons, are supported by the cubic self-attraction in the 2D free space in a two-component system which includes additional linear mixing between the components via the first spatial derivatives, that represent the spin-orbit coupling in binary BEC^12^,^13^,^14^.

Thus, the stabilization of multidimensional fundamental solitons and solitary vortices is an issue of great significance. One possibility to achieve this purpose is to add a self-defocusing quintic term to the self-focusing cubic one^15^,^16^,^17^,^18^. In that model, all fundamental solitons are stable; indeed, the creation of a (2 + 1)D stable optical soliton in a colloidal waveguide, featuring the appropriate cubic-quintic nonlinearity, has been recently reported^19^. However, vortex solitons are stabilized, in the cubic-quintic system, only above a specific threshold, which implies that the vortex rings must be very broad^2^,^15^,^18^, making the experimental realization of such states unfeasible.

The most universal means for the stabilization of fundamental and vortical solitons in 2D and 3D geometries is provided by the use of periodic potentials, as was predicted theoretically in various settings^20^,^21^,^22^,^23^,^24^. Such effective 2D potentials in optical media can be induced by spatially periodic modulations of the refractive index (photonic lattices)^20^. In BEC, similar 2D and 3D potentials are imposed by means of optical lattices, i.e., interference patterns created by pairs of coherent laser beams illuminating the condensate^25^,^26^. In the experiment, photonic-lattice potentials were used to generate stable 2D optical fundamental^27^,^28^ and vortex^29^,^30^,^31^ solitons. A new remarkable experimental result is the creation of 2D plasmon-polariton solitons in microcavities, which are also supported by a lattice structure^32^.

A completely different approach to the creation of self-trapped fundamental and vortical solitary modes was proposed in Refs. 33, 34 and further elaborated in diverse settings^35^,^36^,^37^,^38^,^39^: the self-repulsive nonlinearity, whose local strength in the *D*-dimensional space grows from the center to periphery, as a function of radial coordinate *r*, at any rate faster than *r^D^*, supports extremely robust families of solitons and solitary vortices, along with more complex modes, such as vortex-antivortex hybrids^39^. This format of the nonlinearity modulation can be induced by means of various techniques. In optical media, one may use inhomogeneous density of a nonlinearity-enhancing resonant dopant^40^. In fact, the dopant density may be spatially uniform, while the resonance detuning gradually decreases from the center to periphery under the action of an appropriate external field. In BECs, the extreme tunability of the magnetic Feshbach resonance (FR)^41^ suggests a versatile possibility for the creation of spatially inhomogeneous nonlinearity landscapes by means of properly shaped magnetic fields^42^,^43^,^44^. Furthermore, optically controlled FR^45^, as well as the combined magneto-optical control mechanism^46^, make it possible to create any desirable spatial profiles of the self-repulsive nonlinearity. The required pattern of the laser-field intensity controlling the optically-induced FR can be also "painted" by means of fast-moving laser beams^47^.

A unique property of the model with the spatially growing self-repulsive nonlinearity is the extreme robustness of self-trapped modes in it, and a possibility to create "exotic" modes, which do not exist, or are completely unstable, in usual models, such as the above-mentioned "hybrids". In particular, the application of a strong perpendicular torque to a 3D vortex soliton originally leads to complete destruction ("pulverization") of the mode, which is quickly followed by *spontaneous restoration* of a new vortex, with the axis rotated so as to absorb the total angular momentum.

The objective of this work is to demonstrate another dynamical regime for 2D and 3D systems of this type, which would be impossible in conventional systems: "charging" the ground state, i.e., creation of single- and multiple-vortex structures by the application of a phase modulation in the form of a strong torque to the wave function of the ground state. We demonstrate below that the models based on the self-trapping induced by the spatially modulated self-repulsive nonlinearity, in the absence of any linear potential, exhibit their extreme pattern-forming robustness in these dynamical regimes too: originally, the strong torque completely destroys the ground state, but the resulting "pulverized" configuration quickly re-stabilizes, building one or several vortices, which are displaced from the center and feature stable orbital motion. The application of a strong torque to usual 2D nonlinear systems (the uniform self-repulsive nonlinearity, combined with the isotropic harmonic-oscillator (HO) trapping potential) irreversibly destroys the ground state, replacing it by a turbulent mixture. In 3D, the usual nonlinear systems are still more problematic, since long vortical filaments tend to be unstable, as shown in BEC^48^,^49^ and other settings^50^.

In linear systems, which amount to the Schrödinger equation for the multidimensional HO, the torque-application problem can be solved analytically. It is shown below that the torque fails to generate fundamental vortices with charges *S* = ±1, while vortices with *S* = ±2 are generated with a low efficiency. Thus, unlike the nonlinear system the "charged" HO stays in the ground state, mixed with small contributions of many vortical states with even values of *S*.

## Results

### Formulation of the model and the governing equations

The 2D and 3D settings, in which robust localized modes can be supported by the spatially modulated repulsive cubic nonlinearity in BEC, are modeled by the scaled Gross-Pitaevskii equation (GPE) for the mean-field wave function, *u*(*x,y,z,t*)^33^,^34^,^35^,^36^,^37^,^38^,^39^:
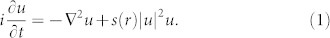
Here *t* is time and *s*(*r*) > 0 is the defocusing-nonlinearity strength that, as said above, must grow along the radial coordinate, *r*, faster than *r^D^*. The 2D version of Eq. (1) finds another physical realization in optics, as the propagation equation (the nonlinear Schrödinger equation) in the spatial domain for the amplitude of the electromagnetic wave in a bulk self-defocusing medium^33^,^34^,^35^,^36^,^37^. In that case, the role of the evolution variable is played by the propagation distance.

Following Refs. 34, 35, 38, 39, we adopt the model with a steep modulation profile, *s*(*r*) = exp(*ar*^2^) in Eq. (1), where α = 0.1 is fixed by rescaling. Accordingly, the asymptotic form of stationary solutions with chemical potential *μ* at *r* → ∞ is
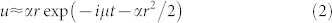
(note that the asymptotic form, on the contrary to that in usual systems, is *universal*, as it does not depend on *μ*, nor on the type of solution – fundamental, vortical, etc.)^34^,^38^,^39^. This steep format of the spatial modulation is not necessary, but it helps to produce basic results in a compact form, although they are not dramatically different from findings for milder modulation profiles, cf^33^,^37^,^38^. Of course, in a real physical situation the nonlinearity coefficient cannot assume extremely large values at large values of *r*. However, this is not necessary, as the solitons supported by Eq. (1) are strongly localized objects, which allows one to truncate the nonlinearity growth at a distance from the center which essentially exceeds the soliton's size.

In terms of cylindrical coordinates, 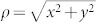
*, θ, z*, stationary states with integer vorticity *S* ≥ 0 have the form of
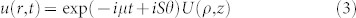
with the real function *U*(*ρ, z*) vanishing as *ρ^S^* at *ρ* → 0 (*z* is absent in the 2D setting). Soliton families are characterized by their norm and angular momentum, which are related in a simple way:
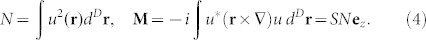


These quantities, along with the Hamiltonian, 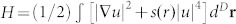, are dynamical invariants of Eq. (1).

To "charge" the ground state, i.e., generate vortices in it, we apply the torque along the *z* direction with strength *p*, and width *W* in the *x* direction, by multiplying the wave function at *t* = 0 with the respective phase-imprinting factor:
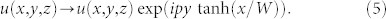


In the experiment, the phase torque can be imposed onto the BEC by illuminating it with a broad laser beam carrying the corresponding transverse phase pattern. In the (2 + 1)D optical system, the same phase pattern may be lent to the soliton-building beam. Note that, while the torque imparts the angular momentum to the condensate, according to Eq. (4), it does not induce a certain integer value of the vorticity; in fact, as shown below, the torque originally induces a mixture of different vorticities.

### Charging the two-dimensional nonlinear system by the torque

**Figure 1:**
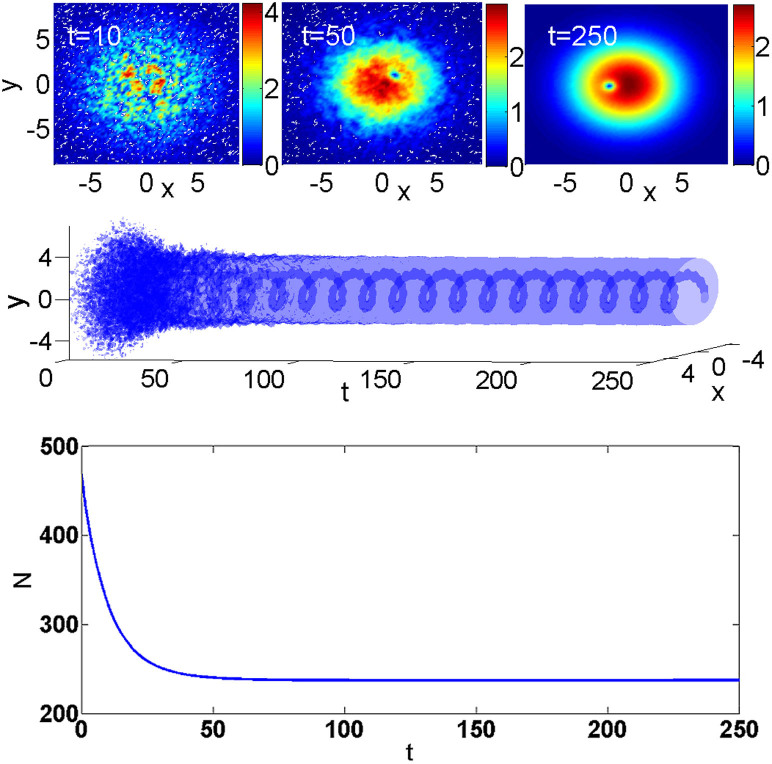
Upper panels: Snapshots of stages of the spontaneous creation of a vortex with a single pivot and *S* = 1 in the 2D setting, by the application of torque (5), with strength *p* = 80 and width *W* = 10, at *t* = 10, 50, and 250. Here, |*u*(*x*,*y*)| is displayed, instead of local density |*u*(x,y)|^2^, for better visibility of the peripheral area. Middle panel: The history of the formation of the orbiting vortex is shown by plotting the intensity-level plot corresponding to |*u*(x,y)|^2^ = 3. Lower panel: The corresponding evolution of the norm. The drop of the norm at the initial stage is caused by elimination of the emitted radiation by the absorber.

The application of a strong torque leads to conspicuous emission of radiation, which should be absorbed, to prevent it from perturbing the dynamics after reflection from the edge. Without the action of the absorber, the violent radiation resulting from the strong torque does not allow the system to converge to a robust state, hence the presence of the absorber is crucially important. After the convergence was reached, the norm and the angular momentum of the localized mode remain virtually constant, and the absorber plays no significant role, see the lower panel in Fig. 1. In the real physical situation with BEC, the role similar to that of the absorber may be played by evaporation of hot atoms from the external trap. In Fig. 1 we present a generic example of the outcome of the application of torque (5) to the ground state of the 2D version of the nonlinear model. The ground state was found by means of the well-known imaginary-time integration method. Originally, a relatively strong torque completely "pulverizes" the trapped state, which is followed by the spontaneous emergence of an eccentrically placed pivot, around which a vortex with *S* = 1 is formed. The self-trapping of the well-organized vortex state from an apparently chaotic one is an example of the above-mentioned ability of the present nonlinear model to (re)build regular structures from a completely destroyed background.

As said above, the simulations were run with an absorber placed at edges of the computation domain (it can model the atomic or photon losses in the experiment, due to a finite size of the setup). Under the action of the absorber, a part of the norm and angular momentum is lost at the initial stage of the evolution, while these quantities remain constant in the course of the subsequent evolution.

Subsequently, the vortical pivot performs stable circular motion around the center, keeping distance *R* = 1.5 from it, with angular velocity *ω* = 0.45. The angular momentum and norm of this established state are *M* = 190.8 and *N* = 237.1, hence the momentum is not sufficient to satisfy relation *M* = *SN* for stationary vortex solitons, see Eq. (4) (hereafter, all values of *M* and *N* refer to a later stage of the evolution, which is no longer affected by the absorber). In fact, this is the reason for obtaining the eccentric vortex orbiting the center, instead of a stationary one pinned to the center.

**Figure 2:**
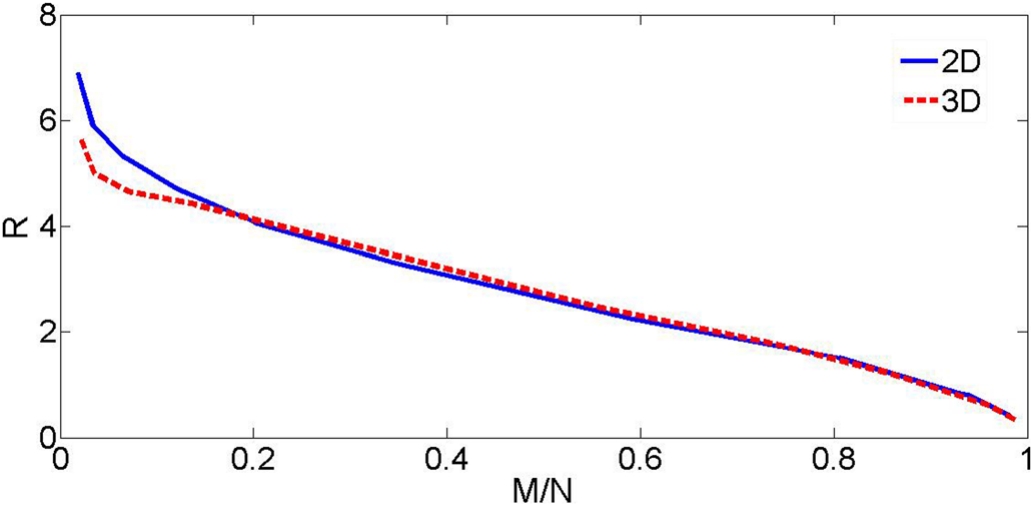
The radius of the circular motion of the vortical pivot around the center, vs. the momentum/norm ratio. The solid blue and dashed red lines pertain to the 2D and 3D models, respectively.

Results of systematic simulations are collected in Fig. 2, which demonstrates that the decrease of *M/N* pushes the pivot farther from the axis. We stress that the curve is universal, representing the results obtained for many different values of the norm and different values of the torque's strength, *p*, in Eq. (5). Thus Fig. 2 shows that the radius of the circular motion of the pivot depends only on ratio *M/N*, rather than on *N* and *M* separately, provided that the single vortex is generated by the torque.

**Figure 3:**
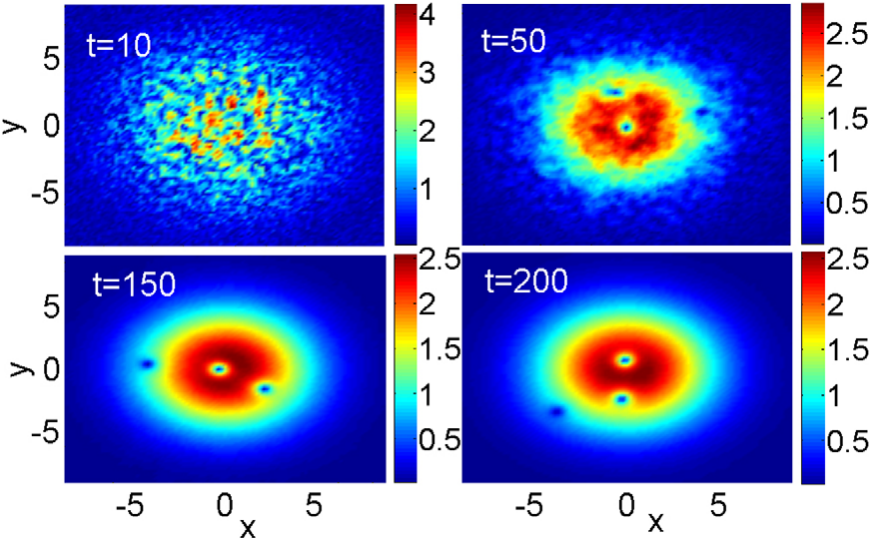
(Color online) Snapshots of the evolution ending up with the formation of the 2D vortex state with three pivots. The pivots which are closest to the center, the middle one, and farthest from the center, perform the orbital motion with angular velocities *ω* = +0.17, −0.44 and −0.48, respectively. Note that, on the contrary to the Keplerian motion, the angular velocity increases with the radius. This is explained by the fact that the centripetal force is provided here not by attraction to the center, but rather by the repulsion from the outer region.

In general, the application of the torque to the ground state can lead to a variety of scenarios following the "pulverization" stage, ranging from restoration of the fundamental soliton to appearance of states with multiple pivots, representing multiple topological charges. These pivots orbit the origin at different angular velocities in clockwise and counterclockwise directions, in some cases colliding with each other. A typical example of the complex (but non-chaotic) dynamical state with three pivots is displayed in Fig. 3, when the torque with *p* = 100 and *W* = 10 was applied. The total angular momentum, is split between the three pivots rotating in opposite directions, is M = 70.5, while the norm of the state is N = 236.4.

It is relevant to mention that steady circulation of a vortex around the center was experimentally observed in a nearly-2D binary BEC^51^. The difference is that in the corresponding setting the confinement was imposed on the two-component system by the trapping potential, while here we consider the dynamics of the single-component self-trapped vortex modes. Nevertheless, the experiment and the present results suggest that the orbital motion of the vortex' pivot around the center is a generic dynamical regime, which occurs in very different systems.

### Charging the three-dimensional nonlinear system by the torque

**Figure 4:**
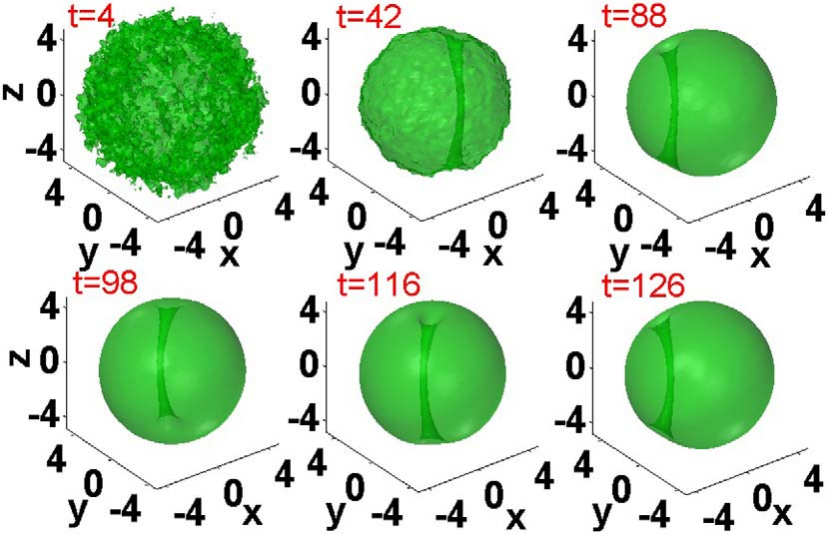
The creation of a single vortex filament, orbiting around the center, by torque (5) with *p* = 40 and *W* = 3 in the 3D setting. Shown are intensity iso-surfaces at |*u*(x,y,z)|^2^ = 1.

In the 3D model based on Eq. (1), stable *vortex filaments* are created by the application of the torque to the ground state, which is represented by the isotropic fundamental soliton. Figure 4 demonstrates an example of the evolution following the application of the initial torque (5) to the ground state, which ends up with norm *N = * 2615.5 and angular momentum (oriented along the *z* axis) *M* = 906.4. After an initial chaotic stage, a vortex filament self-traps, stably orbiting around the center. Thus, the system, this time in its full 3D form, again demonstrates the propensity to spontaneously build robust topologically organized robust structures from a state which was, apparently, completely destroyed by the sudden application of the torque.

**Figure 5:**
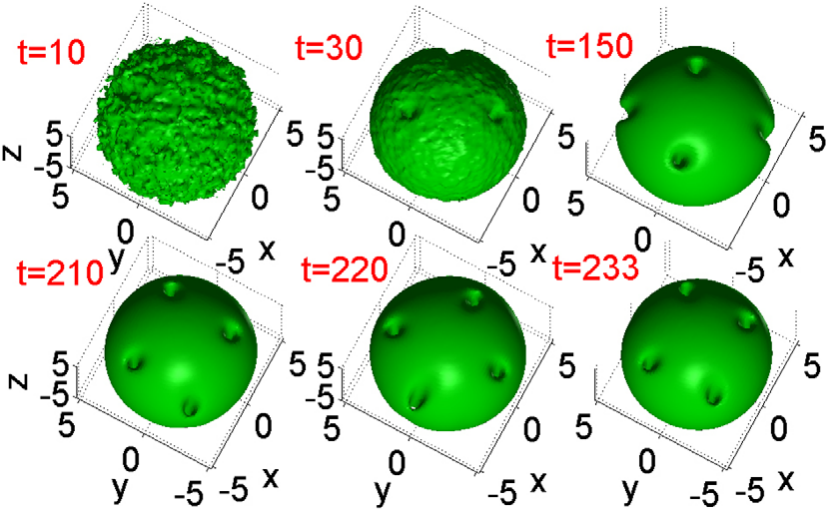
The creation of a "crystallized" complex of four vortex filaments in the 3D setting. Shown are intensity iso-surfaces |*u*(*x*,*y*,*z*)|^2^ = 1. The established complex rotates at angular velocity *ω* = 0.14.

The creation of an array of multiple vortical filaments is also possible in the 3D setting. A drastic difference from the 2D case, where the coexisting vortices move independently (with different angular velocities, see Fig. 3), is that the 3D setting demonstrates a trend to *crystallization* of multiple vortex axes into a steadily rotating complex. This difference is explained by the fact that the third dimension, *z*, enhances the interaction between parallel filaments (the more the system is elongated in *z*, the stronger this interaction is). Figure 5 demonstrates the creation of a four-vortex complex and its stable rotation, with the same norm *N* = 2615.5, as in the case shown in Fig. 5, but with a much larger total angular momentum, *M* = 2650 (which slightly exceeds *N*, cf. Eq. (4)). The panel in Fig. 5 pertaining to *t* = 233 displays the vortex complex rotated by angle π relative to its position at t = 210. Varying the parameters, it is possible to produce rotating complexes with different numbers of the pivots, both smaller and larger than 4.

Besides the well visible holes at the given intensity level, there are also shallow holes that may collide with others. Therefore, multi-hope configuration s feature only transient robustness, which, in some cases, persists for quite long times, allowing the pattern to rotate as a rigid body. Such a case is displayed in Fig. 5, where a robust complex of four visible holes performs more than two full rotation periods.

### Charging the ground state of a linear system by the torque

**Figure 6:**
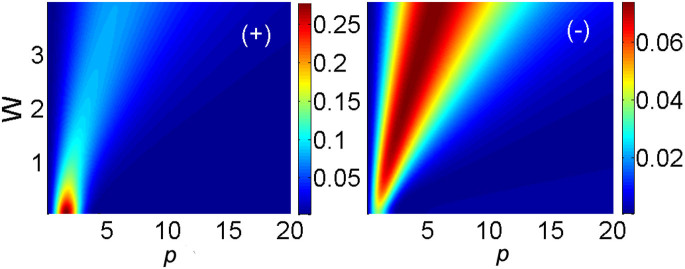
Probabilities of the generation of vorticities with *S* = +2 and −2 [labeled (+) and (−)], |〈*p*,*W*|*S*〉|^2^, in the 3D harmonic oscillator, by the torque, calculated as per Eq. (9) with Ω ≡ 1.

For the sake of comparison with the above results, the creation of the vorticity by the suddenly applied torque is analyzed in the linear system, taking, as a tractable model, the 2D or 3D HO. Although this problem is straightforward to solve, we have not found a solution in the literature, therefore it is presented in a brief form here. The Schrödinger equation for the 3D HO, with trapping frequencies Ω and *χ* in the (*x*,*y*) plane and along the *z* direction, is
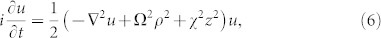
*χ* = 0 corresponding to the 2D HO. The result of the analysis (details are given in section Methods) is that, unlike the nonlinear system considered above, the torque cannot generate states with odd vorticities in the linear model, including *S* = ±1. Even vorticities, starting from *S* = ±2, are generated, but the efficiency is quite poor, as shown in Fig. 6. Thus, the nonlinear system considered above far outperforms its linear counterpart.

## Discussion

The objective of this work is to demonstrate that the recently introduced class of systems, in which extremely robust self-trapping of fundamental and vortical modes are supported by the spatially inhomogeneous self-repulsive cubic nonlinearity, whose local strength grows from the center to periphery, exhibits its exceptional dynamical robustness also in scenarios of "charging" the ground state (imparting the vorticity to it) by the suddenly applied phase torque. It is found that, in both the 2D and 3D versions of the model, the strong torque at first completely destroys the ground state, as it would happen in usual nonlinear systems, but, on the contrary to the usual systems, the present one quickly re-stabilizes by building one or several eccentrically placed vortices, which perform stable orbital motion around the center. An additional advantage of the 3D version of the system is the stability of long vortex filaments. Further, the difference between the 2D and 3D realizations of the system is that in 2D multiple vortices orbit the center independently, while in 3D they tend to crystallize into a structure which rotates as a whole (with residual internal oscillations). We have also demonstrated that the "charging" of the ground state in the linear system (the 3D HO (harmonic oscillator)) by the torque is very inefficient, in comparison with the present model: the linear system cannot generate vorticities *S* = ±1, while probabilities of generating *S* = ±2 are very low.

Thus, the systems with the spatially modulated self-repulsive interactions have a strong potential for the creation of stable multidimensional localized objects, including quite complex ones, which do not exist or are completely unstable in usual settings.

## Methods

The underlying equation (1) was simulated in real and imaginary time alike by the Fourier- transform split-step method in the 3D domain of size (6π)^3^, covered by a mesh of 256^3^ points. As mentioned above, an absorber was installed at edges of the integration domain. Simulations of the 2D model were carried out in a domain of area of (6π)^2^, covered by 256^2^ points. In both the 2D and in 3D cases, the simulations were performed up to *t* = 300. By that time, all dynamical configurations would relax into a final shape.

For the analytical solution of the linear model based on Eq. (6), wave functions with vorticity *S* were taken in the standard form,
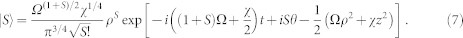
The application of torque (5) to the ground state, which corresponds to *S* = 0 in Eq. (7), gives rise to the following input state:
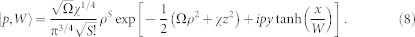
This wave function should be expanded over the full set of eigenstates (7), the respective amplitudes being (hereafter, we simplify the formulas, fixing Ω = 1 by means of rescaling, while *χ* may be arbitrary):
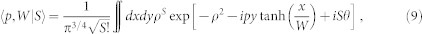
which is written in the mixed Cartesian-polar form. It is easy to see that Eq. (9) yields *c_S_* = 0 for all odd values of *S*. Therefore, the lowest torque-created vorticity is *S = * ±2. Numerically computed values of the respective probabilities, given by integrals (S4), |〈*p*,*W*|*S*〉|^2^, are presented in Fig. 6. The plots demonstrate that, naturally, the probability of the generation of the positive vorticity, *S* = +2, by the positive torque is larger than for *S* = *−*2. The former probability attains a well-pronounced maximum, (|〈*p*,*W*|*S*〉|^2^)_max_ = 0.28 at *W* = 0 and *p* = 1.64 (in the left panel of Fig. 6). A fully analytical expression for the probabilities can be obtained in the limit of *p,W* → ∞, so that *p*_0_ ≡ *p*/*W* is kept constant, and the respective probabilities for *S* = ±2 simplify to
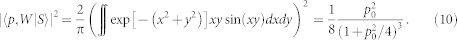
It is easy to check that Eq. (10) agrees with the portions of Fig. 1 corresponding to large values of *p* and *W*. In this connection, it is relevant to mention that, in the limit of *p,W* → ∞, the corresponding torque factor in Eq. (5), exp(*ip*_0_*yx*), carries zero angular momentum, therefore we have |〈*p*,*W*|*S* = +2〉|^2^ = |〈*p*,*W*|*S* = −2〉|^2^ in this limit.

Lastly, it follows from Eq. (10) that, in the same asymptotic limit, the largest probability of generating the vortices with *S* = ±2 is attained at 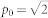, *viz*., 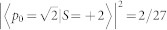. At the same point, 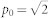, the probability that the HO stays in the ground state (*S* = 0) is 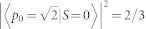. The remaining probability, 1 − 2 × (2/27) − 2/3 = 5/27, is distributed between even higher-order vorticities, |*S*| ≥ 4.

## Author Contributions

All the authors contributed equally to the formulation of the considered problem and drafting the manuscript. R.D. and B.A.M. have performed the numerical and analytical parts of the investigation, respectively. R.D. has produced all figures.
